# Feeding practices and association of fasting and low or hypo glycaemia in severe paediatric illnesses in Malawi – a mixed method study

**DOI:** 10.1186/s12887-020-02305-4

**Published:** 2020-09-04

**Authors:** Fatsani Ngwalangwa, Chawanangwa Mahebere Chirambo, Cecilia Lindsjö, Queen Dube, Josephine Langton, Tim Baker, Helena Hildenwall

**Affiliations:** 1 Department of Paediatrics, College of Medicine, University of Malawi, P/Bag 360, Blantyre, Malawi; 2 MACHI Initiative, P.O Box 30012, Chichiri Blantyre, Malawi; 3 Department of Care Science, Malmö University, 205 06 Malmö, Sweden; 4 Department of Paediatrics, Queen Elizabeth Central Hospital, P.O Box 95, Blantyre, Malawi; 5 Department of Global Public Health, Karolinska Institutet, 171 77 Stockholm, Sweden; 6 Astrid Lindgren Children’s Hospital, Karolinska University Hospital, Stockholm, Sweden; 7 Department of Clinical Science, Intervention and Technology, Karolinska Institutet, 141 52 Huddinge, Sweden

**Keywords:** Fasting, Hypoglycaemia, Feeding, Paediatric illnesses

## Abstract

**Background:**

The presence of low or hypo glycaemia in children upon admission to hospital in low income countries is a marker for poor outcome. Fasting during illness may contribute to low blood glucose and caretakers’ feeding practices during childhood illnesses may thus play a role in the development of low or hypo glycaemia. This study aims to describe the caretaker’s feeding practices and association of fasting with low or hypo glycaemia in sick children in Malawi.

**Methods:**

A mixed method approach was used combining quantitative cross-sectional data for children aged 0–17 years admitted to Queen Elizabeth Central Hospital (QECH), a tertiary hospital in Malawi, with qualitative focus group discussions conducted with caretakers of young children who were previously referred to QECH from the five health centres around QECH. Logistic regression was used to analyse the quantitative data and thematic content analysis was conducted for qualitative data analysis.

**Results:**

Data for 5131 children who were admitted through the hospital’s Paediatric Accident and Emergency Department (A&E) were analysed whereof 2.1% presented with hypoglycaemia (< 2.5 mmol/l) and 6.6% with low glycaemia (≥2.5mmoll/l – < 5 mmol/l). Fasting for more than eight hours was associated with low glycaemia as well as hypoglycaemia with Adjusted Odds Ratios (AOR) of 2.9 (95% Confidence Interval (CI) of 2.3–3.7) and 4.6, (95% CI 3.0–7.0), respectively. Caretakers demonstrated awareness of the importance of feeding during childhood illness and reported intensified feeding attention to sick children but face feeding challenges when illness becomes severe causing them to seek care at a health facility.

**Conclusion:**

Results suggests that caretakers understand the importance of feeding during illness and make efforts to intensify feeding a sick child but challenges occur when illness is severe leading to fasting. Fasting among children admitted to hospitals may serve as a marker of severe illness and determine those at risk of low and hypoglycaemia.

## Background

Hypoglycaemia is a complication of many common illnesses in children in low income countries [1–3] and is also a well-recognised predictor of in-hospital death [4–6]. The World Health Organisation (WHO) currently defines paediatric hypoglycaemia as a blood glucose value below 2.5 mmol/l in a well-nourished child and < 3 mmol/l in a severely malnourished child [7]. Recent studies have reported an increased mortality also in children admitted with low glycaemia, that is blood glucose levels of 2.5–5.0 mmol/l [4, 5, 8]. While the mechanisms of hypoglycaemia in severe illness are not completely clear, multiple potential causes have been proposed whereof fasting has been suggested by some studies to be a potential contributing factor [6, 9–11]. However, these studies only focused on hypoglycaemia and not low glycaemia, which is also associated with increased risk of death among admitted children [4]. Some studies suggest that caretakers may feed children lower quantities of foods and /or less frequently when they are sick [12–14] or restrict feeding during specific illnesses [15]. Substandard feeding practices during paediatric illness could be a possible contributor to low or hypo glycaemia in sick children.

We performed this study to describe caretaker’s feeding practices in sick children in the communities and the potential association between fasting and the presence of low or hypo glycaemia in sick children in Malawi.

## Methods

### Study design

This is a parallel mixed method study combining cross-sectional data on reported fasting hours and the presence of low or hypoglycaemia among severely ill children being admitted to Queen Elizabeth Central Hospital (QECH), a tertiary hospital in Malawi, with focus group discussions conducted with caretakers of young children in five catchment areas of health centres around QECH and were previously referred to QECH from those respective health centres due to severe illnesses. The mixed methodology was deemed appropriate to get a deeper understanding of the community feeding circumstances that could possibly lead to fasting and low or hypo glycaemia during childhood illnesses.

### Setting

QECH is located in the city of Blantyre and is the largest referral hospital in Malawi with 1000 inpatient beds and 180 peadiatric inpatient beds. Patients are referred mainly from the district hospitals in the southern region of Malawi and from health centers in Blantyre district. Patients are referred from health centers within Blantyre directly to QECH emergency department if they have severe illness or require specialised care. All referred patients and reasons for referral are documented in the health centre registers. Health Surveillance Assistants (HAS) are located within communities but report to specific allocated health centres. Five health centres in Blantyre (Zingwangwa, Limbe, Mpemba, Mlambe and Ndirande) were selected for qualitative data collection. Two focus group discussions were conducted in rural areas, two in urban areas and one in the semi urban areas. The paediatric department at QECH serves 100,000 children annually with approximately 23,000 admissions annually. All children reporting to QECH are seen at the Paediatric Accident and Emergency (A&E) Department where they are triaged to different rooms depending on the disease severity. Children with a WHO emergency sign [16], are identified during triage and immediately transferred into the resuscitation room for treatment and stabilisation. All severely ill children being treated in the resuscitation room have vital signs recorded and bed side blood glucose test done. An average of 300 children are admitted via the resuscitation room every month.

### Study participants

The study included all children aged 0 to 17 years who were triaged as severely ill upon arrival to QECH and consequently assessed and treated in the resuscitation room of QECH. A severely ill child was any child with a WHO emergency sign [16]. The qualitative component included caretakers of young children in five catchment areas of health centres around QECH who had also experienced referral to QECH due to severe illness from the respective health centres. The FGDs were conducted in rural, urban and semi-urban population.

### Data collection

All children treated in the resuscitation room at the paediatric A&E have their clinical and demographic data recorded. Information collected included reported hours of fasting and admission blood glucose values as well as demographic data like age and sex, vital signs, nutritional status, admission WHO emergency sign(s), whether the patient was referred from another health facility. Blood sugar tests were conducted using capillary blood drawn from a fingure prick analysed in a Hemocue Glucose 201RT point of care analyzer, manufactured by HemoCue AB, Sweden. The glucometer uses cuvettes to draw blood directly from a fingure prick, then it is placed into the machine to give a reading within a minute. Quality control checks of the glucometers were performed weekly using GlucoTrol-NG control fluids. Data from all patients treated in the resuscitation room from December 2016 to January 2019 was anonymised for analysis.

Mothers of young children who were previously referred from the five selected health centres to QECH due to severe illness were identified through Health Surveillance Assistants (HSAs) to participate in Focus Group Discussions (FGDs) about their feeding practices during childhood illnesses. Five FGDs consisting of 6 to 11 mothers who had experienced care-seeking for a severely ill child at QECH were conducted at the beginning of the quantitative data collection in December 2016. The FGD participants were asked open ended questions regarding their feeding practices for a sick child. The FGD facilitator probed for any preference and non-preference for specific types or amounts of food, and specific conditions that could impact the feeding and other challenges related to the feeding of sick children. A discussion topic guide was prepared in advance and the facilitator probed to get a deeper understanding of views from participants. A pilot FGD was conducted, questions in the topic guide were modified for clarity and the order of the question was changed. The FGDs were conducted in Chichewa and lasted for 1.5 to 2 hours. A social scientist (CMC) experienced in qualitative research and fluent in English and Chichewa facilitated the FGDs with the assistance of a Chichewa speaking note taker who documented any non-verbal communication. The interviews were recorded using a digital audio recorder. The facilitator transcribed and translated interviews where-after random back translation checks were performed to confirm accuracy.

### Data analysis

Stata version 15 was used to analyse the quantitative data for all children who had been assessed in the resuscitation room. Hypoglycaemia was defined according to the WHO definition of < 2.5 mmol/l or < 3 mmol/l in severely malnourished children and low glycaemia was defined as a blood sugar of 2.5 - < 5.0 mmol/l. A reported fasting time of more than eight hours was considered as prolonged fasting. Proportions, means or median were used as appropriate to describe the baseline characteristics. Multinomial logistic regression was used to analyse the association between reported fasting and low or hypo glycaemia. Multivariate logistic regression was used to adjust for confounding and the following variables were used in the multivariate analysis; fasting, age, sex, severe acute malnutrition as diagnosed by the admitting clinician, referral from another facility and the presence of any WHO emergency signs [16]. Severe Acute Malnutrition was based on the clinical diagnosis of the admitting team and this was based on clinical signs: the presence of visible severe wasting, bilateral pitting oedema and/or mid upper arm circumference (MUAC) < 11.5 cm or < 70% of weight for height for an average child of the same age as per local and WHO guidelines [17, 18]. The variables in multivariate analysis were pre-identified by the study investigators based on their clinical plausibility. All observation with missing blood glucose and fasting hours were removed from the analysis.

Thematic content analysis was used to analyze the qualitative data. A deductive approach was used to code the data and generate themes related to the objectives of the study. The transcripts were read thoroughly by the principle investigator to gain deeper understanding of the data. Then, line to line manual coding was done by two researchers (FN and HH). Codes with the same meaning were bundled into categories and later grouped into themes. The whole process of qualitative data analysis was done manually.

## Results

### Baseline characteristics

A total of 5273 children were assessed and treated in the resuscitation room at QECH between December 2016 to January 2019. Data for 5131 participants were analysed due to missing recordings of participant’s blood sugar levels or fasting hours for 142 (2.7%) children. The median age of the included children was 1.8 years (interquartile range 0.6–5.0). A total of 58.8% were males. Severe acute malnutrition was present in 336 (6.6%) of the study population. A quarter of patients (*n* = 1314, 25.6%) had been fasting for more than eight hours prior to assessment at the hospital (Table 1). The majority of patients had a blood sugar of 5.0 mmol/l or higher (*n* = 4685, 91.3%) while 336 (6.6%) had low glycaemia and 107 (2.1%) had hypoglycaemia. (Table 1).

**Table 1:** Baseline Characteristics of 5131 children admitted at the Accident and Emergency Room, Queen Elizabeth Central Hospital

Variable	All	Normo-glycaemia (> 5 mmol/l)	Low-glycaemia (2.5 - < 5 mmol/l)	Hypoglycaemia (< 2.5 mmol/l)
	***N*** = 5131	***N*** = 4685	***N*** = 339	***N*** = 107
	N (%)	N (%)	N (%)	N (%)
**Age**				
< 1 month	309 (6.0)	270 (5.8)	33 (9.7)	6 (5.6)
≥ 1 month- < 1 year	1536 (29.9)	1463 (31.2)	54 (15.9)	19 (17.8)
≥ 1 yr- 5 yrs	1934 (37.7)	1709 (36.1)	172 (50.7)	53 (49.5)
> 5 yrs	1352 (26.4)	1243 (26.5)	80 (23.6)	29 (27.1)
Median years (IQR)	1.8 (0.6–5.0)	1.7 (0.6–5.0)	2.6 (1.1–5.0)	2.5 (0.9–4.6)
**Fasting hours**				
≥ 8 h	1314 (25.6)	1087 (23.2)	164 (48.4)	63 (58.9)
**Sex**				
Female	2114 (41.2)	1929 (41.2)	136 (40.1)	49 (45.8)
**WHO emergency sign**				
Present	3493 (68.1)	3204 (68.4)	207 (61.1)	82 (76.6)
**Severe Malnutrition**				
Yes	336 (6.6)	268 (5.7)	46 (13.6)	22 (20.7)
**Referred from other facility**				
Yes	3946 (76.9)	3571 (76.2)	283 (83.5)	92 (86.0)

WHO emergency sign present if any of the following obstructed breathing, severe respiratory distress, cyanosis, coma, convulsion, shock and severe dehydration

### Association of fasting and other admission factors with low/hypoglycaemia

Reported fasting time of more than eight hours was associated with an increased risk of low glycaemia with an Unadjusted Odds Ratio (UOR) of 3.1 (95% Confidence interval (CI) 2.5–3.9). The UOR for hypoglycaemia and fasting was 4.7 (95% CI 3.2–7.0). When adjusted for age, severe malnutrition, sex, presence of an emergency signs as defined by the World Health Organisation and referral from another facility the Adjusted Odds Ratio (AOR) for low glycaemia after more than eight hours of fasting was 2.9 (95% CI 2.3–3.7) and for hypoglycaemia 4.6 (95% CI 3.0–7.0).

Age of less than one month (AOR 3.0 (95% CI 2.0–4.9), age between one year to less than five years (AOR 1.8, 95% CI 1.3–2.3) presence of severe acute malnutrition (AOR 2.9, 95% CI 2.0–4.1) and being referred from another facility (AOR 1.5, 95% CI 1.1–2.0) were associated with the presence of low glycaemia whilst severe acute malnutrition (AOR 5.0, (95% CI 3.0–8.2) was associated with hypoglycaemia. The presence of a WHO emergency sign was associated with a reduced risk of low glycaemia (AOR 0.7, 95% CI 0.6–0.9) but increased risk of hypoglycaemia (AOR 1.6, 95% CI 1.1–2.6). (Tables 2 and 3).

**Table 2:** The association between fasting, other admission factors and low glycaemia

**Variable**	**UOR**	**95% CI**	***P*-value**	**AOR**^**a**^	**95% CI**	***P*-value**
**Fasting hours**						
≥ 8 h	3.1	2.5–3.9	< 0.001	2.9	2.3–3.7	< 0.001
**Age (ref > 5 yrs)**	1.0					
**<** 1 month	1.9	1.3–2.9	0.002	3.0	2.0–4.9	< 0.001
≥ 1 month- < 1 yrs	0.6	0.4–8.3	0.003	0.8	0.6–1.2	0.381
**≥** 1 yr- ≤ 5 yrs	1.6	1.2–2.1	0.001	1.8	1.3–2.3	< 0.001
**Sex**						
Female	1.0	0.8–1.2	0.703	0.9	0.7–1.2	0.533
**WHO Emergency signs**						
Present	0.7	0.5–0.9	0.005	0.7	0.6–0.9	0.018
**Malnutrition**						
Present	2.5	1.9–3.6	< 0.001	2.9	2.0–4.1	< 0.001
**Referred from other facility**						
Yes	1.6	1.2–2.1	0.002	1.5	1.1–2.0	0.005

^a^Adjusted for fasting and all the covariates in the table

WHO emergency sign present if any of the following obstructed breathing, severe respiratory distress, cyanosis, coma, convulsion, shock and severe dehydration

*UOR* Unadjusted odds ratio, *AOR* Adjusted odds ratio, *CI* Confidence interval

**Table 3:** The association between fasting, other admission factors and hypoglycaemia

Variable	UOR	95% CI	***P***-value	AOR^a^	95% CI	***P***-value
**Fasting hours**						
≥ 8 h	4.7	3.2–7.0	< 0.001	4.6	3.0–7.0	< 0.001
**Age (ref > 5 yrs)**						
< 1 month	1.0	0.4–2.3	0.915	1.4	0.6–3.6	0.416
≥ 1 month- < 1 yrs	0.6	0.3–0.9	0.049	0.8	0.4–1.5	0.424
≥ 1 yr- ≤ 5 yrs	1.3	0.8–2.1	0.193	1.3	0.8–2.2	0.216
**Sex**						
Female	1.2	0.8–1.8	0.338	1.2	0.8–1.8	0.385
**WHO emergency signs**						
Present	1.5	1.0–2.4	0.071	1.6	1.1–2.6	0.036
**Malnutrition**						
Present	4.1	2.5–6.6	< 0.001	5.0	3.0–8.2	< 0.001
**Referred from other facility**						
Yes	1.9	1.1–2.3	0.021	1.7	1.0–3.0	0.062

^a^ Adjusted for Fasting and all the covariates in the table. WHO emergency sign present if any of the following obstructed breathing, severe respiratory distress, cyanosis, coma, convulsion, shock and severe dehydration

### Parental feeding practices in child illness and reasons for fasting

Three themes emerged from the focus group discussion interviews 1) Decreasing appetite with increasing disease severity 2) Caretakers trying their best to feed sick children 3) Food not to give during illnesses and reasons why.

#### Decreasing appetite with increasing disease severity

Caretakers mentioned malaria, pneumonia, diarrhoea and fever to be common childhood illnesses and that certain illnesses like diarrhoea, vomiting and malaria can affect the appetite and consequently the feeding in children. However, some caretakers reported that a variety of illnesses can cause lack of appetite and poor feeding such that it is generally not easy to feed a sick child.

*“In general, when you are sick, many diseases take away the appetite indicating that things are not working normally in the body”* (ZR4, FGD5)

Some caretakers reported that it is the disease severity that affects the appetite and feeding pattern of a sick child such that the more severe the disease the more difficult it is to feed a sick child regardless of the type of the disease.

“*All diseases when they come strong, food does not taste nice”* (NR3, FDG4)

*“It depends on the illness that has caught the child, when too serious, it is hard for them to eat”* (MR2, FGD2)

#### Caretakers trying their best to feed a child when the child is sick

Caretakers emphasised the importance of continued feeding of children during illnesses. It was mentioned that it is the responsibility of caretakers to ensure that the child is properly fed during illness.

*“When a child is sick, it’s not easy to feed the child because the appetite is mostly gone. But it is up to us mothers to make sure the child eats, no matter what*” (MR8, FGD 2*)*

Caretakers expressed some preference of the types of food they wanted to provide to their sick children. They chose to provide food that they know will provide energy and extra nutrients to a sick child.

“*We prepare porridge we add necessities in there so that when the child eats should get some nutrients”* (MR8, FGD2)

Some provided food of the child’s preference because they knew that the child would eat even though they were sick. Some caretakers believed that they should feed porridge to a child who is vomiting because porridge was perceived to remain in the body even after the child had vomited

*“We try so hard to find nice food stuff that are not even taken under normal circumstances”* (NR6, FGD 4)

Some caretakers reported a change in the feeding pattern, they reported an increase in the frequency of feeding to ensure that the child has eaten enough food. Some also said that they would force their children to eat during illness.

*“We try to do everything for her, even if it means holding the mouth for her to eat” (R3, FGD1)*

However, some caretakers admitted that there are times when a child completely refuses to eat any food and in that case some chose to take the child to the hospital because they believe that the hospital provides intravenous drips to give a sick child some strength.

*“(when the child is completely refusing to eat any food) is the point where you rush to the hospital. Since health workers would know how to handle it. When they see something like that they quickly put a ‘drip of water’. After a ‘drip of water’ they do know that the child could gain strength again. When it comes to that there is little we could do as parents it is only health workers who would know what to be done. That part is for the health workers” (*ZR 5, FGD 5)

Caretakers expressed concerns for the potential consequences of poor feeding that they said could present as a lack of sugar, lack of energy, inadequate amounts of blood in the body, malnutrition and low levels of water.

*“If the child is refusing food then that would create other diseases like malnutrition, frequent sicknesses, lack of blood, body salts, sugars since there are a lot of foods that we eat and those foods contribute to the body and when is refusing then there is no contribution. So that would create problems since there is no food working in his body”.* (ZR6 FGD5)

#### Food not to give during illnesses and reasons why

Some caretakers believed that there are certain types of food that should not be given to sick children; food containing sodium bicarbonate, fizzy drinks should not be fed to sick children. Others reported that fizzy drinks could increase the disease severity.

*“I would like to add that it is not right to give fizzy drinks. When a child is taking medication and it mixes with fizzy drinks it creates poison in the body. And there are no nutrients in fizzy drinks that would help the child. They are just useless water to the body”* (ZR4, FGD5)

They also mentioned that there are other food types that they do not give to sick children because they believe some of them increase the severity of the disease and they do not add any nutrients to the body. Others believed that certain food types disturb the function of the medicine the child is taking during illnesses

“*Do not give porridge with soda, lemons and milk because they disturb the functioning of the medicine*” (R5, FGD1)

## Discussion

This study shows that fasting for more than eight hours is associated with an increased risk of low and hypo glycaemia among severely sick children presenting to hospital in a resource limited setting. Still, Malawian caretakers demonstrated awareness of the importance of feeding during childhood illness such that they reported to change the pattern, the food preferences and feeding attention given to children when sick but they face challenges when the child has severe illness and is refusing to feed.

Increased glucose demand during illness [19] coupled with illness induced anorexia [20] and subsequently poor feeding [21] may possibly contribute to the increased risk to develop low and hypo glycaemia among sick children, hence the importance of adequate feeding during illness must be emphasized [22]. Fasting has been suggested to be associated with an increased risk of hypoglycaemia in children in earlier studies [2, 9, 23]. This study supports these findings with results from a large sample of severely ill children on admission irrespective of underlying diseases. We also demonstrate an association with low glycaemia, possibly suggesting that low glycaemia constitute a transient state that develops into hypoglycaemia as severity of illness and poor feeding progress. Both low and hypo glycaemia are known risk factors for mortality among sick children [1, 4] hence early and continuous feeding may help to minimise the development of low and hypo glycaemia and subsequently mortality in sick children.

The relationship between illness severity and feeding problems was acknowledged in focus group discussions, where caretakers reasoned that any disease if presentation is severe can affect and diminish the feeding. While plausible that fasting and the associated low blood glucose levels are simply markers of disease severity as intake commonly declines with increased illness severity [24, 25] we found no association of WHO emergency signs with low glycaemia and a weak association between WHO emergency signs and hypo glycaemia. Indeed, the presence of fasting in a sick child was already used by health workers as a proxy for hypoglycaemia in the absence of diagnostic testing in a study on current management of paediatric hypoglycaemia among Malawian health workers [26].

Caretakers expressed an understanding of the importance of feeding during childhood illnesses and they declared that adequate feeding of a sick child is a responsibility of the caretaker. This is in contrast to other studies that have suggested that mothers may restrict or even withhold food during illness [27] due to beliefs that the illness may ‘disturb’ the digestive system [28] or traditional beliefs that children benefit from not being fed during illnesses [15, 29]. The overall response by caretakers in this study was to intensify feeding attempts of sick children and to feed them more frequently with the food of the child’s preference. This shows an awareness by the community on the importance of feeding during childhood illnesses as stipulated in the WHO Global strategy for infant and young child feeding [30].

Still, caretakers noted that severely ill children are difficult to feed and they expressed situations of helplessness when a child refuses to feed such that they would then resort to seek hospital care. The Integrated Management of Childhood Illness (IMCI) guidelines recognizes poor feeding as a danger sign and recommends referral to higher level facilities [31]. Improved stabilisation strategies for sick children at lower level facilities and minimised delays in referral could reduce the occurrence of low or hypo glycaemia and poor outcomes in severely sick children. The association between poor feeding and low and hypo glycaemia could also be due to presence of underlying malnutrition which leads to a vicious cycle of increased frequent infections and poor feeding [32]. Malnutrition is common in low income settings [33] and has shown to be associated with both low and hypo glycaemia in this study.

The strength of this study is that the quantitative findings were triangulated by the qualitative information to highlight the caretaker’s actions surrounding feeding in childhood illness. Other strengths include the use of a large sample of sick children that provided more robust results on the relation between fasting and low blood glucose values and the inclusion of children across all paediatric age groups. A limitation is that the FGDs were only conducted with caretakers in the communities; FGDs conducted with caretakers at the hospital would have brought a closer relationship with fasting in the study population and also a more recent experience on feeding during illness. However, from an ethical point of view, we felt it was more appropriate to invite FGDs participants who were not currently suffering the stress of having a severely sick child admitted at hospital. The FGD participants from the community had also experienced previous care-seeking at QECH for a severely ill child and were thought to give a more nuanced view of feeding challenges given some time had passed since their child was sick. Another limitation of the study is that information on treatment received before referral to QECH was not captured and consequently some participants might have received intravenous fluids including dextrose before arrival to QECH underestimating the effect of pre-admission fasting on hypo/low glycaemia. Furthermore, the classification of Severe Acute Malnutrition relied on the admitting clinicians to appropriately use the clinical definitions in the guidelines and this might have led to some cases of misclassification.

## Conclusion

Malawian caretakers understand the importance of feeding during illness and make efforts to feed children when sick. Severe illness pose challenges in the feeding practices leading to fasting. Fasting for more than eight hours is a risk factor for low and hypo glycaemia which are known risk factors for poor outcome. Health workers need to be aware of the risk of low and hypoglycaemia among severely ill children. Fasting in sick children presenting to hospitals may serve as a marker of disease severity and low or hypoglycaemia.

## Data Availability

The datasets used and analysed during the current study are available from the corresponding author on reasonable request.

## Abbreviations

QECH: Queen Elizabeth Central Hospital
A&E: Accident and Emergency
CI: Confidence Interval
OR: Odds Ratio
AOR: Adjusted Odds Ratio
UOR: Unadjusted Odds Ratio
WHO: World Health Organisation
HSA: Health Surveillance Officer
FGD: Focus Group Discussion
IQR: Interquartile Range
N: Number
